# Does Reactive Thrombocytosis Observed in Iron Deficiency Anemia Affect Plasma Viscosity?

**DOI:** 10.5505/tjh.2012.13008

**Published:** 2012-10-05

**Authors:** Selami K Toprak, İbrahim Tek, Sema Karakuş, Nihat Gök, Nazmiye Kurşun

**Affiliations:** 1 Başkent University, School of Medicine, Department of Hematology, Ankara, Turkey; 2 Medicana International Ankara Hospital Cancer Center, Ankara, Turkey; 3 Ankara University, School of Medicine, Department of Biostatistics, Ankara, Turkey

**Keywords:** Iron deficiency anemia, Reactive thrombocytosis, Thrombocythemia, Plasma viscosity

## Abstract

**Objective:** The accompanying thrombocytosis is referred to as the major factor associated with thromboembolism in iron deficiency anemia (IDA). Increased viscosity may increase the risk of thrombosis. We hypothesized that increased platelet count -with reactive thrombocytosis- might also affect plasma viscosity. We planned to evaluate the influence of normal and high platelet count on plasma viscosity in IDA patients.

**Material and Methods:** The patient population consisted of fifty-three newly diagnosed and untreated women aged between 18 and 62 years with IDA. Group 1 consisted of 33 patients, platelet levels below 400 x 10^9^/L. Group 2 consisted of 20 patients, platelet levels above 400 x 10^9^/L. Measurements of plasma viscosity were performed using Brookfield viscometer.

**Results:** Mean plasma viscosity was found as 1.05 ± 0.08 mPa.s. in Group 1, and 1.03 ± 0.06 mPa.s. in Group 2. Mean plasma viscosity was not statistically different. White blood cell count was significantly higher in Group 2. Vitamin B12 levels were significantly higher in Group 2, while folic acid levels were higher in Group 1 (p=0.011 and p=0.033). Plasma viscosity was correlated with erythrocyte sedimentation rate (r=0.512 p=0.002) in Group 1 and inversely correlated with vitamin B12 (r=−0.480 p=0.032) in Group 2.

**Conclusion:** Despite the significant difference between groups in terms of platelet count, no significant difference was detected in plasma viscosity and this finding could be explained as the following; 1-These platelets were not thrombocythemic platelets; 2-Similar to the theory about leukocytes, higher platelet counts – even non-thrombocythemic – may increase plasma viscosity; 3-Evaluating platelet count alone is not sufficient and the associating red-cell deformability should also be taken into account; and 4-Although other diseases that could affect viscosity are excluded, some definitely proven literature criteria such as fibrinogen, hyperlipidemia, and the inflammatory process should also be evaluated by laboratory and clinical measures.

## Abstract (tr)

**Amaç:** Demir eksikliği anemisine eşlik eden trombositoz, gelişebilecek tromboembolizm için en önemli etken olarak görülmektedir. Artmış viskozite, tromboz riskini de arttırabilir. Buradan hareketle, trombosit sayısındaki artışın da-reaktif trombositoz- plazma viskozitesini etkileyebileceği varsayımında bulunduk. Demir eksikliği anemisi bulunan hastalarda, normal ve yüksek trombosit sayısının, plazma viskozitesi üzerindeki etkisini değerlendirmeyi amaçladık.

**Gereç ve Yöntemler:** Yakın zamanda demir eksikliği anemisi tanısı alıp, daha herhangi bir tedavi başlanmayan ve yaşları 18 ile 62 arasında değişen 53 kadın hasta çalışmaya dahil edilmiştir. Grup 1, trombosit sayısı < 400 x 10^9^/L olan 33 hastadan oluşmaktadır. Grup 2, trombosit sayısı > 400 x 10^9^/L olan 20 hastadan oluşmaktadır. Plazma viskozite ölçümleri, Brookfield viscometer kullanılarak yapılmıştır.

**Bulgular:** Ortalama plazma viskozitesi, Grup 1’de 1.05 ± 0.08 mPa.s. ve Grup 2’de ise 1.03 ± 0.06 mPa.s. olarak bulunmuştur. İki grup arasında, ortalama plazma viskozitesinde istatistiksel bir fark yoktur. Grup 2’de lökosit sayısı anlamlı olarak yüksek bulunmuştur. Grup 1’de folik asid ve Grup 2’de ise vitamin B12 düzeyleri, diğer gruba göre anlamlı olarak yüksek saptanmıştır (p=0.011 ve p=0.033). Plazma viskozitesi, Grup 1’de eritrosit sedimentasyon hızıyla doğru (r=0.512 p=0.002) ve Grup 2’de ise vitamin B12 düzeyi ile ters korelasyon göstermektedir (r=−0.480 p=0.032).

**Sonuç:** Her iki grup arasında trombosit sayısı bakımından anlamlı bir fark olmasına karşın, plazma viskozitesinde istatistiksel bir fark bulunamamıştır. Bunun nedenleri olarak şunlar sayılabilir: 1- Bu trombositler, “trombositemik” özellik taşımamaktadırlar. 2- Lökositlerle alakalı teoriye benzer şekilde, belki de ancak çok yüksek trombosit sayısı -trombositemik olmasalar bile- plazma viskozitesini arttırabilmektedir. 3- Sadece trombosit sayısını değerlendirmek yeterli olmamakta, ek olarak eritrositlerin yapısal özellikleri de hesaba katılmalıdır. 4- Viskoziteyi etkileyebilecek başka hastalıkların dışlanmasına karşın, fibrinojen, hiperlipidemi ve enflamatuvar sürecin de laboratuvar ve klinik olarak değerlendirilmesi uygun olacaktır.

## INTRODUCTION

Thrombocytosis is frequently encountered as a coincidental laboratory finding. The causes of thrombocytosis, in which the platelet count exceeds the upper limit can be categorized as 1- reactive or secondary due to infections, trauma, surgery, iron deficiency (ID), or occult malignancy; 2- clonal, including essential thrombocythemia (ET) and other myeloproliferative disorders; and 3- familial [1]. The clinical features of secondary thrombocytosis are almost always a result of the underlying disorder provoking the reaction. Even though thrombocytosis is benign and self-limiting in most cases and virtually absent in reactive thrombocytosis -unless provoked by other features-, this disorder can result in hemorrhage or thrombosis [2,3]. The accompanying thrombocytosis is frequently referred to as the major factor associated with thrombembolism in iron deficiency anemia (IDA) [4]. Plasma viscosity is known to have a close relationship with blood flow. Increased viscosity may increase the risk of thrombosis or thromboembolic events [5]. Many factors can affect plasma (and/or blood) viscosity, such as white blood cell and platelet count, hematocrit, immunoglobulins, and fibrinogen. We hypothesized that increased platelet count -with reactive thrombocytosis- might also affect plasma viscosity In the present study, we planned to evaluate the influenceof normal and high platelet count on plasma viscosityin IDA patients.

## MATERIALS AND METHODS

**Study Population **

The patient population consisted of fifty-three newly diagnosed and untreated women aged between 18 and 62 years with a demonstrable cause of IDA. There were two groups. Group 1 consisted of 33 patients with IDA and platelet levels below 400x10^9^/L. Group 2 consisted of 20 patients with IDA and platelet levels above 400x10^9^/L. Patient characteristics are summarized in Table 1. 

Menorrhagia was the most frequent cause of IDA in both groups. Patients possessing chronic renal failure, hypertension, coronary vascular disease, diabetes mellitus, cigarette addiction, hyperlipidemia, coagulopathy, acute blood loss, infectious disease, connective tissue disorders, anemia of chronic disease, and cancer were excluded from the study. Group 2 patients are evaluated for primary thrombocytosis and other causes of reactive thrombocytosis and those with other disorders causing reactive thrombocytosis were also excluded. 

The study protocol was approved by the local ethics committee and written and signed informed consent was provided by all participants. 

**Methods**

IDA was diagnosed based on hemoglobin (Hb), red blood cell indexes, serum iron, serum iron binding capacity, serum ferritin, and transferrin saturation test results, as previously reported [6,7,8]. Thrombocytosis was defined as platelet count ≥400x10^9^/L in at least two blood samples. 

To measure plasma viscosity, 5 cc of blood samples were collected from patients into anticoagulated tubes. All samples were frozen because immediate measurement of plasma viscosity was not possible and the results of fresh frozen samples and freshly studied blood samples were the same. By this method, all samples were studied at the same time and errors that could be due to calibration of the test machine were minimized. Blood samples taken into anticoagulated tubes were first centrifuged at 3000 rpm for 5 min and then the separated plasma was frozen at −40°C. On the day of measurement, all samples were melted and recentrifuged and then measured at 37°C in a Brookfield DV– II + Cone Plate Viscometer (Brookfield, Stoughton, MA, USA) machine, which was calibrated with distilled water. Each sample was measured four times and the average of the measurements was taken. Some sources indicate that the normal value of plasma viscosity is between 1.3 and 1.65 mPa.s., while others state that it is 1.10-1.30 mPa.s. at 37°C and independent of age and gender [9,10]. 

**Statistical Analysis**

Variables are presented as mean±SD. Analyses of variance was performed to test the difference between two groups with respect to viscosity and the other laboratory measurements according to their platelet count using Ttest and Mann-Whitney U test. The Pearson’s Correlation was used to describe a correlation between independent parameters. A P values less than 0.05 were considered to be statistically significant. All statistical analyses were conducted using SPSS v.15.0 software (SPSS, Inc., Chicago, IL, USA).

## RESULTS

[Table t1]The mean platelet count in Group 1 was 277.90x10^9^/L (range: 155-368x10^9^/L). The mean platelet count in Group 2 was 507.45x10^9^/L (range: 415-645x10^9^/L) (p<0.001). 

Mean plasma viscosity was found as 1.05±0.08 mPa.s. in Group 1, and 1.03±0.06 mPa.s. in Group 2, (normal 1.39±0.08) [9]. The mean plasma viscosity was not statistically different. Table 2 shows the mean of different variables in 53 patients. 

Although there was no significant difference between the two groups in terms of hemoglobin (Hb) and even serum iron, serum iron binding capacity, serum ferritin, and transferrin saturation, levels of mean corpuscular hemoglobin (MCH) and mean corpuscular volume (MCV) were significantly lower in Group 2 (p=0.015 and p<0.001, respectively). In terms of excluding any associating acute or chronic condition that could affect viscosity, it is important that the C-reactive protein (CRP) and erythrocyte sedimentation rate (ESR) levels were within the normal range in both groups (Table 2). However, the white blood cell (WBC) count, which is known to affect viscosity, was significantly higher in Group 2. 

Vitamin B12 levels were significantly higher in Group 2, while folic acid levels were significantly higher in Group 1 (p=0.011 and p=0.033, respectively). 

**Figure 1:**
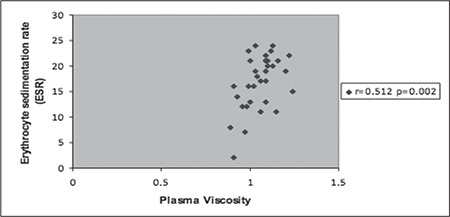
Correlation of plasma viscosity with erythrocyte sedimentationrate in Group 1

**Figure 2:**
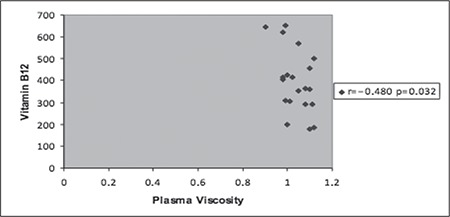
Correlation of plasma viscosity with vitamin B12 inGroup 2

Plasma viscosity was significantly positively correlated with ESR (r=0.512 p=0.002) in Group 1 and inversely correlated with vitamin B12 (r=−0.480 p=0.032) in Group 2 (Figure 1, Figure 2). Platelet count, mean platelet volume (MPV), WBC count, Hb level, red blood cell indexes, iron indexes, and the others variables such as thyroid stimulating hormone (TSH), CRP and folic acid levels were not associated with plasma viscosity in both groups.

## DISCUSSION

Markers of platelet activation and haemorrheological indices have been demonstrated to play a role in the pathophysiology of atherosclerosis and cardiovascular events through thrombosis [11]. Compared to primary thrombocytosis such as that caused by essential thrombocythemia, reactive thrombocytosis is generally regarded as benign [3]. Nevertheless, reactive thrombocytosis has been reported to cause severe and even lethal complications [3, 12]. The mechanism of this possible association between reactive thrombocytosis-iron deficiency and thrombosis is unknown. Thus we also wanted to investigate whether or not the reactive thrombocytosis]]. Nevertheless, reactive thrombocytosis has been reported to cause severe and even lethal complications [3, 12]. The mechanism of this possible association between reactive thrombocytosis-iron deficiency and thrombosis is unknown. Thus we also wanted to investigate whether or not the reactive thrombocytosis, the process of triggering thrombosis occurs by a possible increase in plasma viscosity. We defined the lower limit of thrombocytosis as 400x10^9^/L and when we compared the plasma viscosity of the 20 patients in the group with thrombocytosis with the other group, no significant difference could be detected. In a study consisting of 113 patients with diagnosed iron deficiency anemia, pancytopenia, polycythemia vera, essential thrombocythemia, idiopathic thrombocytopenic purpura, myelodysplastic syndrome, aplastic anemia, and thalassemia, thrombocytosis –along with many associating variables – had a positive effect on blood viscosity while not affecting plasma viscosity in parallel with our study [5]. Similarly, in another study comparing 20 cases with splenectomy (15 trauma related, 4 idiopathic thrombocytopenic purpura, 1 splenic cyst) to healthy controls, although the difference in thrombocyte count between groups was statistically significant, no significant difference could be detected in plasma viscosity [13]. In this same study, apart from the absence of a significant correlation between viscosity and thrombocyte count, it was reported that the rapidly increasing thrombocyte count following splenectomy returned to normal in the following weeks while the increase in plasma viscosity persisted. The researchers stated that the most important factor here was the decreased red-cell deformability following splenectomy. In the study of Rozenberg et al., patients with history of myocardial infarct were compared with healthy controls and no significant correlation could be detected between blood viscosity and thrombocyte count [14]. The concentration of red cells is the major factor determining the viscosity of normal blood [15]. Leukocytes have little effect because of their relatively small numbers. When the leukocyte count is greatly elevated, changes in viscosity of blood have been noted [15]. WBC count was increased in Group 2, however, it is meaningful that this alone did not affect plasma viscosity. In Ho’s study, similar to increased thrombocyte count, increased WBC count influenced blood viscosity while no significant relation could be detected in plasma viscosity [5]. Some authors suggest that for the leukocyte count be able to affect both the whole blood and plasma viscosity, the granulocyte component should be increased, along with the total WBC count reaching almost 50x10^9^/L [15]. 

There was not a correlation between MCH, MCV and plasma viscosity in both groups; but levels of MCH and MCV were significantly lower in Group 2. The lower levels of these markers in the group with higher platelet count could be explained by a longer and deeper iron depletion in this group. 

Folic acid and vitamin B12 levels were within the normal levels, however, the folic acid level in Group 1 and the vitamin B12 level in Group 2 were significantly higher than the other group and this showed that they did not affect plasma viscosity. Nonetheless, serum vitamin levels in Group 2 were inversely correlated with plasma viscosity (Figure 2). Remacha et al. compared 326 patients with thrombosis to 351 control cases and reported that serum vitamin B12 levels were remarkably decreased in the group with thrombosis [16]. The researchers stated that this was related to the hyperhomocysteinemia, which was significantly different in the patient group. It was demonstrated that high plasma homocystein levels were positively correlated with viscosity both in patients with coronary arterial disease and controls [17]. Thus, although homocystein levels were not measured in the present study, the relation between low B12 levels and high viscosity is meaningful.

In Group 1, the correlation between ESR and plasma viscosity was an expected result [10]. However, it is doubtful why this same tendency could not be observed in Group 2. It was reported that ESR was positively correlated with plasma viscosity in case groups with pulmonary tuberculosis and malignant lymphomas [18]. In this same study, no correlation could be detected between ESR and plasma viscosity in the sickle cell anemia group and healthy controls and furthermore, ESR was remarkably lower in patients with sickle cell anemia when compared with controls. When the more pronounced effect of red cell deformability on plasma viscosity is taken into account and the fact that this parameter is not tested in the patient groups is considered, the non-correlation between ESR and viscosity in Group 2, in which the platelet count is higher than Group 1, can be explained. 

As a result; in contrast to normal platelets in healthy individuals, the circulating thrombocythemic platelets (e.g. chronic myeloproliferative disorders) spontaneously activate and secrete their products, thus forming aggregates that transiently plug the microcirculation or result in occlusive platelet thrombi in vessels [19]. In patients with thrombocythemia associated with chronic myeloproliferative disorders, increased hematocrit and viscosity aggravate the platelet-mediated microvascular ischemic and thrombotic syndrome of thrombocythemia to major arterial and venous thrombotic complications. In the present study, despite the significant difference between groups in terms of platelet count, no significant difference was detected in plasma viscosity and this finding could be explained as the following; 1-these platelets were not thrombocythemic platelets; 2-similar to the theory about leukocytes, much higher platelet counts (e.g. >1.000x10^9^/L) than observed in Group 2 (mean: 507.45x10^9^/L) – even non-thrombocythemic– may increase plasma viscosity; 3-evaluating platelet count alone is not sufficient and the associating red-cell deformability should also be taken into account; and 4-although other diseases that could affect viscosity are excluded, some definitely proven literature criteria such as fibrinogen, hyperlipidemia, and the inflammatory process should also be evaluated by laboratory and clinical measures. We hope that further studies performed on larger number of patients -reactive thrombocytosis- and controls, additionally including thrombocythemic disorders, with the possibility of encountering higher platelet counts and analyzing additional criteria that could influence viscosity can illuminate the dark spots of current clinical practice in reactive thrombocytosis which seems innocent at the present.

## Figures and Tables

**Table 1 t1:**
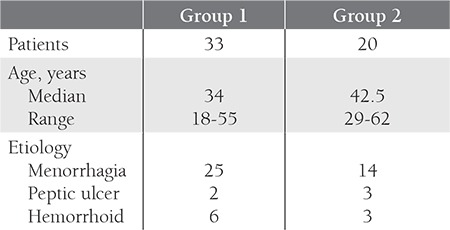
Patient characteristics

**Table 2:**
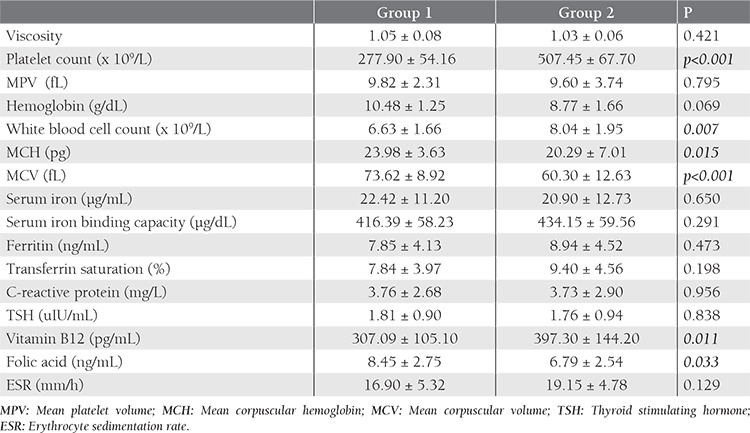
Mean of different variables in two groups
